# Applications of Graphene-Modified Electrodes in Microbial Fuel Cells

**DOI:** 10.3390/ma9100807

**Published:** 2016-09-29

**Authors:** Fei Yu, Chengxian Wang, Jie Ma

**Affiliations:** 1 School of Chemical and Environmental Engineering, Shanghai Institute of Technology, 100 Hai Quan Road, Shanghai 201418, China; fyu@vip.163.com (F.Y.); wang3359@163.com (C.W.); 2 State Key Laboratory of Pollution Control and Resource Reuse, School of Environmental Science and Engineering, Tongji University, 1239 Siping Road, Shanghai 200092, China

**Keywords:** graphene, electrode, microbial fuel cell, anode, cathode

## Abstract

Graphene-modified materials have captured increasing attention for energy applications due to their superior physical and chemical properties, which can significantly enhance the electricity generation performance of microbial fuel cells (MFC). In this review, several typical synthesis methods of graphene-modified electrodes, such as graphite oxide reduction methods, self-assembly methods, and chemical vapor deposition, are summarized. According to the different functions of the graphene-modified materials in the MFC anode and cathode chambers, a series of design concepts for MFC electrodes are assembled, e.g., enhancing the biocompatibility and improving the extracellular electron transfer efficiency for anode electrodes and increasing the active sites and strengthening the reduction pathway for cathode electrodes. In spite of the challenges of MFC electrodes, graphene-modified electrodes are promising for MFC development to address the reduction in efficiency brought about by organic waste by converting it into electrical energy.

## 1. Introduction

Although fossil fuels are essential for economic development, the increasing consumption of fossil fuels has come with significant negative drawbacks, such as air pollution and global warming, which have consequently accelerated the exploration of renewable energy technologies by scientists [1,2]. The microbial fuel cell (MFC) is a promising recently developed device that can convert the chemical energy stored in organic fuels as nutritional substrates into electrical energy though the metabolism of microorganisms, while degrading the organic contaminant to an extent [3,4,5]. Compared with traditional chemical fuel cells [6], large-scale organic substrates, such as municipal treatment plants [7,8], agriculture wastes, solid wastes from dairy farms [9,10,11,12], and even human waste [13,14], can be used as fuels in MFCs. However, many factors affect the performance of MFCs, including the chemical substrate, ionic concentration, proton exchange material, catalyst, internal resistance, electrode spacing, and electrode materials [15,16,17,18,19,20]. The low extracellular electron transfer (EET) efficiency between the microorganism and the electrode is still the main bottleneck limiting the practical applications of MFCs, resulting in poor energy conversion efficiency and low power density [21]. Generally, there are two main approaches used to cope with these problems: one is to improve the electrode properties by surface treatment [22], for example, by means of microbial reduction [23], electrostatic incorporation or ionic liquid functionalization [21]; the other is to fabricate new electrode materials to enhance the EET at the anode or the catalytic activity at the cathode [22].

Because microbial growth on metal surfaces can accelerate metallic corrosion in an aqueous environment [24], carbon-based materials, such as carbon cloth [3], carbon paper [25], carbon felt [26], carbon fiber [27] and graphite particles [4,28], are used as electrode materials for MFCs. With the rapid developments in materials research, graphene, a new and renowned member of the carbon family, has been adopted in MFC electrodes because of its excellent physical and chemical properties, for instance, its high specific surface area (2630 m^2^·g^−1^) [29,30,31], outstanding electrical conductivity [32], and extraordinary biocompatibility [33]. Additionally, it has been widely used in Li-ion batteries [34], supercapacitors [35,36], sensors [37], electrochemical catalysts [38], and oil sorbents [39].

Graphene, discovered in 2004 by Geim and Novoselov [40], is a two-dimensional (2D) single-atom-thick flat material consisting of *sp*^2^ hybridized carbon atoms arranged in a honeycomb lattice [41,42,43]; at present, graphene is the thinnest material in the world. Its theoretical thickness value, band length, and bond angle are 0.335 nm, 0.142 nm, and 120°, respectively [44]. However, the monolayer graphene sheet is known to irreversibly agglomerate or form multilayer graphite through strong π-π stacking and van der Waals interactions [45]. Graphene oxide (GO) is an important derivative of graphene that contains heavy epoxy and hydroxyl functional groups on the basal planes, and carbonyl and carboxyl groups on the sheet edges, which make it possible to fabricate graphene based materials on a large scale [42]. Although these functional groups increase its hydrophilic character, the conjugated *sp*^2^ network of the individual graphene basal planes is disrupted and the electrical properties of GO are decreased. Therefore, the removal of oxygen functional groups can enhance the electrical conductivity of graphene modified materials.

Compared with 2D graphene, three-dimensional (3D) graphene structures have outstanding characteristics, e.g., a large accessible surface, excellent mechanical strength, and remarkable flexibility [46], which can enhance the number of microorganisms on their surface and are the ideal electrode materials in MFCs. Recently, different reduction methods have been developed to obtain macroscopic 3D graphene structures from GO sheets, e.g., hydrothermal reduction, chemical vapor deposition, chemical reduction, electrochemical reduction, and microbial reduction [47]. In this review, the methodologies on the synthesis of graphene-based electrodes, and the design principles of a desirable MFC electrode are covered. The influence of graphene-modified electrodes (anodes and cathodes) on the electricity generation of MFCs is analyzed and discussed.

## 2. Synthesis Methods of Graphene-Modified Electrodes

Due to the appealing properties and outstanding structures of graphene, significant effort has been devoted to the fabrication of graphene-modified electrodes to enhance the EET between the anode materials and microbes at the anodes, and the catalytic activity at the cathodes. GO can be synthesized from graphite using the modified Hummers method, and it always functions as a precursor to prepare graphene and its composites in many studies. The different types of synthesis methods for graphene-modified electrodes in MFCs are summarized in this section.

### 2.1. Oxidization and Reduction Methods

The oxidization and reduction method is a promising method for the mass production of graphene and graphene-based composites from graphite. The hydrophobic graphite powder can be changed to hydrophilic graphite oxide under the influence of strong oxidants, e.g., concentrated sulfuric acid and potassium permanganate, which break the π bond between the graphite sheets and also introduce abundant epoxy, hydroxyl, and carbonyl groups [48]. However, the hydrophilic functional groups introduced disrupt the conjugated *sp*^2^ network and degrade the electrical properties of the graphite sheets [42]. Hence, to enhance the electrical properties, first, the graphite oxide solution is homogeneously dispersed by ultrasonic treatment to form a graphene oxide colloidal solution, and then reducing agents are introduced to remove the oxygen functional groups, resulting in a reduced graphene oxide (rGO). Hydrazine hydrate, hydroquinone, sodium borohydride, and hydrogen sulfide have been extensively explored as reducing agents. The advantages of the graphite oxide reduction method include its low cost, short production time, simple operation, and capability for large-scale production. More importantly, there are many ways to reduce GO, e.g., hydrothermal reduction [4,49], chemical reduction [50,51,52,53], electrochemical reduction [54,55,56,57], solvothermal reduction [22,58,59], and microbial reduction [23,60]. On the contrary, the low quality, reduced conductivity, and large defect population of rGO limit the application of these methods. More seriously, the toxicity of some of these reducing agents is a significant disadvantage.

**Figure 1:**
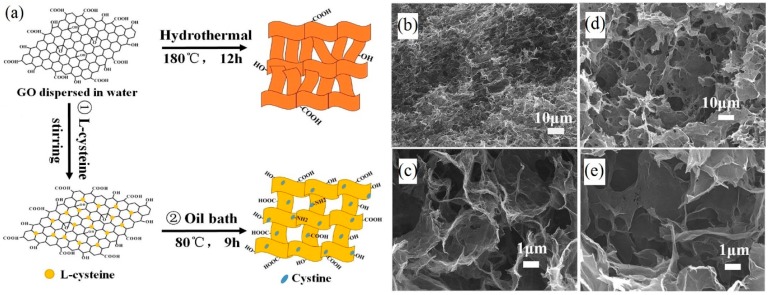
(**a**) Schematic illustration of the chemical reduction and hydrothermal methods. Scanning electron microscopy (SEM) images of graphene aerogel (GA) without l-cysteine (**b**,**c**). SEM images of GA with l-cysteine (**d**,**e**). Adapted from [4], with permission from © 2015 The Royal Society of Chemistry.

Qiao et al. [4] used l-cysteine as a reductant to reduce a GO dispersion at a low temperature (80 °C) in an oil bath for 9 h via a chemical reduction method (Figure 1a). 3D graphene hydrogel (GH) is gelated from a solution of 2D GO sheets and freeze-dried for 24 h to convert it into graphene aerogel (GA). Meanwhile, rGO was also obtained from the GO dispersion, without any reductant, using the hydrothermal method in a 50 mL Teflon-lined stainless steel autoclave heated at 180 °C for 12 h. The pore size increased with an increasing ratio of l-cysteine to graphene. When the ratio was 1:13, the pore size maintained a uniform porous structure (Figure 1d,e). The porous structure breaks up when the ratio is over 1:13. In addition, the introduction of l-cysteine apparently resulted in an enlargement of the pore size of GA for the hydrothermal method (Figure 1b,c).

To enhance the biocompatibility of MFC electrodes, Zhuang et al. [60] replaced the depleted medium with a fresh medium containing 1 mL of the GO solution while the MFC had reached a steady state for electricity generation. When the solution of the cathode reactor turned black, it was replaced with a fresh medium without GO. Similarly, Yuan et al. [23] utilized the microbial reduction method to reduce the GO solution in the anode chamber. These bio-electrodes exhibited stronger electrochemical responses, higher EET efficiency, and improved biocompatibility, which is promising for MFC applications in bioenergy generation.

### 2.2. Self-Assembly Methods

Self-assembly is a technology where the basic structural units in solution, e.g., molecules, nanomaterials, and micron or larger scale substances, can spontaneously form an ordered and stable structure; it is one of the most common methods of obtaining 3D graphene (3DG) from GO sheets in homogeneous solutions via the gelation process [46]. In the self-assembly process, the basal planes of GO sheets spontaneously aggregate due to the increased attractive interactions of the van der Waals forces, the hydrogen bond and the π-π stacking interaction from the carbon framework, and the weaker electrostatic interactions from the ionization of the oxygen-containing groups, resulting in an ideal construction of GH [4,61,62]. The gelation of the suspension of GO sheets can be triggered by many methods, such as by adjusting the pH value of the GO solution [50], adding crosslinking agents [63] or using chemical reduction methods [64]. There are many ways to synthesize graphene sheets from the suspension of GO sheets by electrostatic interaction using different self-assembly mechanisms; these include the layer-by-layer assembly method [64,65], the template-induced assembly method [66], hydrothermal treatment [67], and direct freeze-drying [68]. In addition, the number of graphene layers and the pore size can be effectively controlled by changing the experimental conditions, such as the template style, pH value, and concentration of the suspension of GO sheets.

The layer-by-layer assembly method is the main self-assembly technique to synthesize uniform nanostructure films and it relies on the electrostatic adsorption between oppositely charged species. Guo et al. [64] introduced carboxyl groups on the surface of carbon paper by a functionalization process, the carbon paper was then immersed alternately into a positively charged polyethyleneimine (PEI) aqueous solution (10 mg·L^−1^) and a negatively charged graphene suspension for 20 min, and the electrode was washed with deionized water and dried in hot air. This process was repeated several times, resulting in the creation of a layer-by layer film, which improved the electron transfer ability of the blank carbon paper anode because of the increased specific surface area and increased attachment by bacteria.

**Figure 2:**
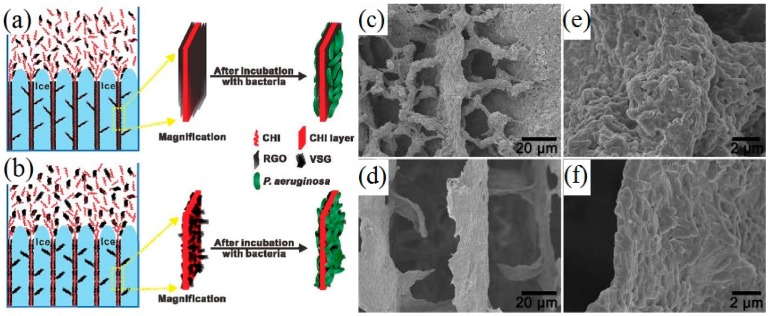
Schematic illustration of the layered and branched structure formation process of chitosan/reduced graphene oxide (CHI/rGO) (**a**) and chitosan/vacuum-stripped graphene (CHI/VSG) (**b**) scaffolds; SEM images of CHI/VSG (**c**,**e**) and CHI/rGO (**d**,**f**) scaffolds after incubation with bacteria at a low (**c**,**d**) and high (**e**,**f**) magnification. Adapted from [68], with permission from © 2012 American Chemical Society.

Ice-segregation-induced self-assembly (ISISA) is a freeze-casting technique that can produce a series of aligned macroporous or layered materials through a commonly used bottom-up approach. He et al. [68] prepared vacuum-stripped graphene (VSG) under vacuum and heated it at 250 °C for 5 min. 5 mL homogeneous chitosan (CHI) solutions with amounts of the VSG powder were drawn into injection syringes, dipped into a liquid nitrogen bath at a constant dipping rate of 5 mm·min^−1^, and then freeze-dried to form CHI/VSG scaffolds (Figure 2b). The macroporous CHI/VSG scaffold formed a thick and multilayered biofilm (Figure 2c,e). On the other hand, the scaffolds prepared with rGO (noted as CHI/rGO) formed a single-layer biofilm (Figure 2d,f), indicating that the biocompatibility of this type of electrode is significantly enhanced as a result of the larger surface roughness of the CHI/VSG scaffolds. In addition, the pore size of the graphene sponges can be easily controlled by the growth rate of ice crystals.

### 2.3. Chemical Vapor Deposition

Chemical vapor deposition (CVD) is a widely applied technique for manufacturing semiconductor films, where the chemical reaction of a carbon source (e.g., methane [69], ethanol [70], and cyanuric chloride [71]) is conducted in a high-temperature, high gas flow rate condition and the resultant film is deposited on the surface of a heated solid substrate. The main substrates used in graphene production are transition metal materials, e.g., Cu [72] and Ni [7]. The graphene films obtained can be transferred to other substrates, maintaining their excellent conductivity and transmittance. Large-area and high-quality graphene can be synthesized through this process [2], but the high cost and complex nature of the process limits its use in large-scale applications. Worse still, the quality of the graphene produced is highly dependent on the substrate.

Yong et al. [70] used a clean nickel foam as the substrate and introduced ethanol as the carbon source into a tube by bubbling a H_2_/Ar gas mixture through an ethanol liquid during a 20 min CVD process. The nickel substrates obtained were etched away with HCl solution (3 M) at 80 °C to form free-standing 3D graphene foams (GFs). Polyaniline (PANI) was then deposited on the surface of the 3D GFs. In a similar manner, Kirubaharan et al. [2] used a plasma-enhanced CVD technique at 1050 °C and at a 10 torr pressure for 1 min to reduce GO sheets in a quartz tube, and they formed the resultant N-doped graphene nanosheets (NG), whose specific surface area was 579 m^2^·g^−1^.

### 2.4. Template Methods

In general, template methods are combined with the other methods mentioned above, and modified electrodes are usually prepared using the soft- or hard-template methods with numerous groups as precursors [73]. The template methods can provide a limited reaction space for growing graphene sheets. The soft-templates are micelle aggregated by a surface active agent, providing a dynamic balance cavity [74], and the hard-templates are solid templates, e.g., nanotubes, porous anodic alumina, and nickel foams [24], providing a series of static pore canals through which the reductants enter the inside of the template [75]. The template methods have many advantages, such as their low cost, the environment-friendly nature, the ease for mass production, and the simplicity of the process; these methods are extensively applied in graphene-modified electrodes.

**Figure 3:**
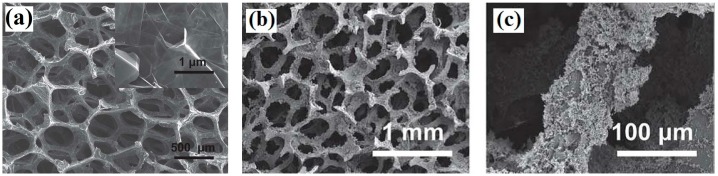
(**a**) SEM image of the graphene sponge (GS) showing the macroscale porous structure and the graphene surface (inset); (**b**,**c**) SEM images of a colonized graphene-sponge-stainless steel mesh electrode (GMS) after 50 days of operation, at different scales. Adapted from [66], with permission from © 2012 The Royal Society of Chemistry.

Krishnamurthy et al. [24] chose nickel foams as a model to prepare a conformal graphene coating on a Ni surface via template-directed CVD, which can prevent the dissolution of Ni from microbial byproducts (e.g., H^+^), though numerous microbial colonies adhere to the nickel foam coated by graphene (Ni/G). Similarly, Xie et al. [66] used a polyurethane sponge substrate to prepare graphene sponge composites (GS) via the dipping-and-drying process in graphene ink. The macroscale porous substrates provided an open 3D structure that increased microbial colonization (Figure 3a–c). The use of a stainless steel mesh inserted in two pieces of the graphene sponge to form a graphene-sponge-stainless steel mesh electrode (GSM) greatly reduced the resistance of the composite electrode from ~180 Ω (GS electrode) to ~22 Ω (GSM electrode).

### 2.5. Other Synthesis Methods

**Figure 4:**
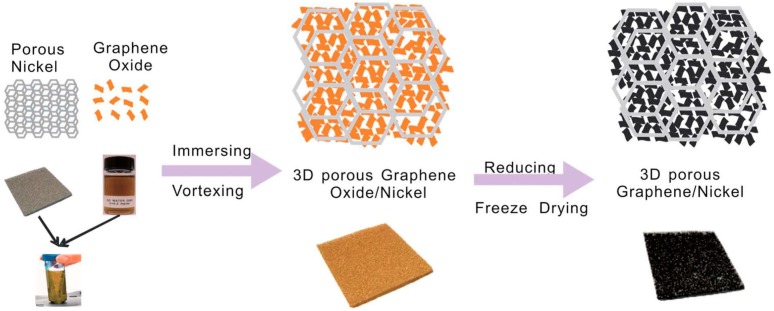
Schematic illustration of the fabrication process for the hierarchical porous graphene/nickel composite electrode. Adapted from [78], with permission from © 2014 The Royal Society of Chemistry.

There are many types of preparation methods that can be used to synthesize graphene-modified electrodes in addition to the four methods above, e.g., the spraying technique [76], the explosion method [71], the ammonia-evaporation-induced method [77], the electrophoresis method [55], electrostatic interaction [21], and the bio-catalytic oxidation process [23]. To investigate the improvement of electricity generation in MFCs, one or more methods may be used in the production of the electrodes. Qiao et al. [78] prepared a GO colloid from graphite using a modified Hummers method. A piece of nickel foam was then immersed in the GO colloid suspension. The resultant graphene oxide/nickel foam composite film was immersed into an ascorbic acid solution overnight to obtain a graphene/nickel foam composite film, and the composite film was subsequently freeze-dried for 24 h. The resultant electrode is referred to as a 3D porous graphene/nickel electrode (Figure 4).

## 3. Development of Graphene-Modified Electrodes in Microbial Fuel Cells

**Figure 5:**
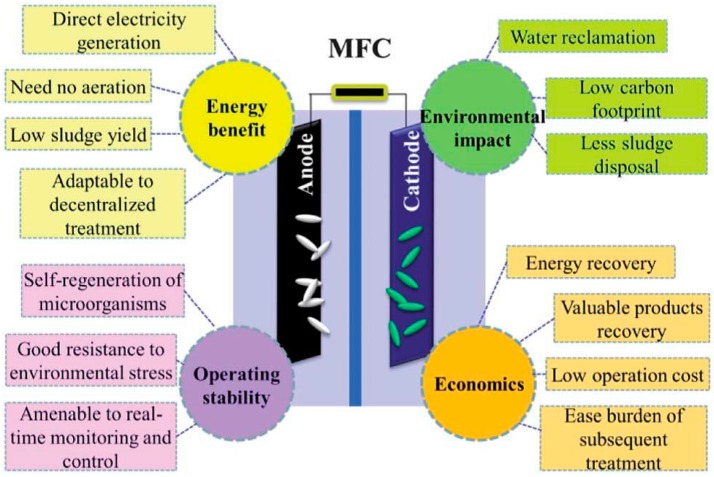
Potential benefits of MFCs for energy, environmental, operational and economic sustainability. Adapted from [81], with permission from © 2014 The Royal Society of Chemistry.

There are two typical reactor configurations used in MFC studies. One is the double-chamber reactor, and the other is the single-chamber reactor [24]. They all consist of two electrodes and an ion exchange membrane, which separates the anode chamber from the cathode chamber. To evaluate the operation of MFC, we observe the open circuit voltage (OCV), electrode potential, internal resistance, power density, current density, coulombic efficiency, and removal efficiency of pollutants. Among them, a higher OCV value is representative of a higher reaction rate [79,80], and the power density is dependent on the EET efficiency, the active area of electrodes, the reaction kinetics, etc. The OCV and the power density are the important parameters for evaluating the operating condition of the MFC. Although MFC research is still in its infancy, the unique advantages of MFCs have drawn the attention of many scientists, and efforts are being made to study various aspects, such as their low environmental impact, large energy benefit, positive societal impact, and stable operation (Figure 5) [81,82]. Moreover, graphene-modified materials have shown improved performance, as mentioned above, which provides a good reason for the integration of graphene-modified materials and MFCs. In this section, the design and applications of graphene-modified electrodes in MFC are reviewed in detail.

### 3.1. Design and Development of Graphene-Modified Anode Materials in MFCs

Due to the exoelectrogens attached on the surface of anode electrodes, the anode materials should not only have good electrical conductivity, anti-corrosive qualities and excellent chemical stability but also outstanding biocompatibility and EET efficiency. Generally, the nutrient solutions of the MFC anode chamber are always inoculated with *Escherichia coli* [2,18,67], *Shewanella oneidensis* MR-1 [54,58,70], *Pseudomonas aeruginosa* [55,83], and anaerobic sludge [23,49,60], which are all anaerobic bacteria. To prepare excellent bio-anodes, a series of factors should be taken into consideration, such as the specific surface area, the biocompatibility, the EET efficiency, and the mechanical properties.

#### 3.1.1. Specific Surface Area

**Figure 6:**
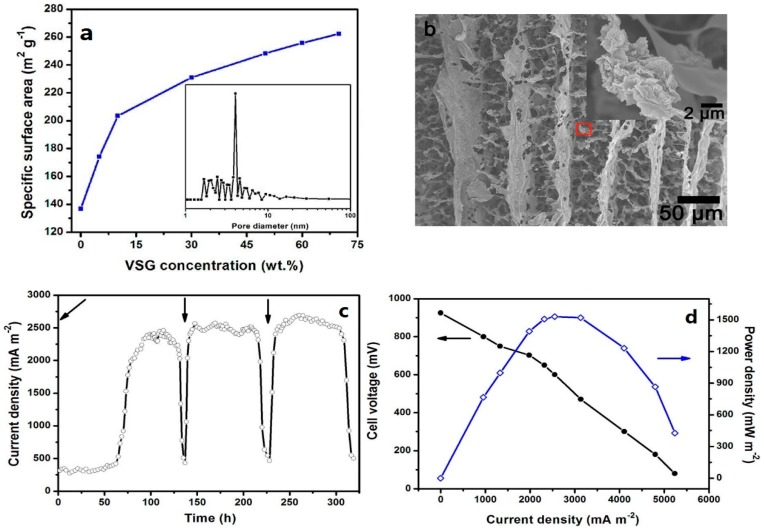
(**a**) The plot of the specific surface area increasing with the vacuum-stripped graphene concentration of the chitosan/vacuum-stripped graphene (CHI/VSG) scaffolds; (**b**) CHI/VSG-50 scaffold; Constant-load discharge curve (**c**); and power density and polarization curves (**d**) of the microbial fuel cells based on the CHI/VSG-50 anode. Arrows of (**c**) indicate the time of glucose feeding. Adapted from [68], with permission from © 2012 American Chemical Society.

While most biological reactions occur on the surface of the anodes, a higher specific surface area of the anode electrodes provides more space for microorganisms to attach to the anode. In general, a high specific surface area always reflects two aspects: one is the obvious hierarchical structure (Figure 2c,d), Figure 6b, and the other is the porous structure (Figure 1d,e). The hierarchical structure exhibited excellent flexibility, which allowed it to be restored to its original shape without any damage when it was compressed to half its volume [3]. Based on the size of the pores, the structures can be divided into micropore, mesopore, and macropore structures. The bacteria growing on the surface of the electrode scaffolds mainly relied on the macropore structure to enhance the interaction between the bacteria and electrodes; the micropore and mesopore structures can improve the EET efficiency on the graphene surface [68].

To control the specific surface area, He et al. [68] obtained a series of CHI/VSG structures by adding different concentrations of VSG via ISISA. The specific surface area increased with an increasing concentration of VSG (Figure 6a). However, when the VSG concentration exceeded 70 wt %, the CHI/VSG scaffolds could be broken. The optimal concentration of VSG was 50 wt % (CHI/VSG-50), whose remarkable maximum power density was 1530 mW·m^−2^, a value that was 78 times higher than that of carbon cloth anodes (19.5 mW·m^−2^). Chen et al. [3] introduced different freezing rates to control the size of the ice templates used during the growth. To prepare GS, GH ice crystals were grown in a small temperature gradient at a low freezing rate in a refrigerator (−10 °C), forming a large ice template. The Brunauer–Emmett–Teller (BET) specific surface area of the GS formed was 55.4 m^2^·g^−1^, which was larger than that for the GF formed at a quick freezing rate in liquid nitrogen (19.9 m^2^·g^−1^). The maximum power density of GS was 0.710 mW·m^−2^ (compared with 0.476 mW·m^−2^ of GF). In addition, the specific surface area of the crumpled graphene layers with a disordered structure was 579 m^2^·g^−1^, approximately 1200 times larger than that of the anode based on graphite (approximately 0.5 m^2^·g^−1^) [2,84], and the maximum power density could become as high as 2668 mW·m^−2^, which increased by approximately 18 times compared with that of the control groups (142 mW·m^−2^). The graphene-modified electrode maintained the excellent property of a large specific surface area, and numerous microorganisms resided on the macropores, which significantly enhanced the electricity generation performance of the MFC. To an extent, the large specific surface area is also suitable for the cathode electrodes to increase the active sites.

#### 3.1.2. Biocompatibility

Due to the biological reaction that occurs on the surface of the anode electrodes, the biocompatibility of anode electrode materials is essential in determining the power output of MFCs [85,86,87]. The biocompatibility of electrode materials can be summarized via two aspects. One is the shape, size and surface roughness of the materials, and the toxic residues in the manufacturing process of these materials is the key factor affecting the biocompatibility [88]. The other is the microbial corrosion effect on these materials [24,89]. To enhance the biocompatibility of anode electrodes, some surface treatment procedures should be applied, such as heat [90], ammonia [91,92] and acid treatments [21,64], and electrochemical oxidation [93]. A cell viability measurement is always the dominant method used to evaluate the biocompatibility of electrode materials [33] by counting the colony-forming units (CFUs) on the surface of the electrodes [94]. Generally, the electrode materials are taken out of the anode chamber after the MFC operated for ~60 h, and then they are tested by counting the viable bacteria growing on the surface of the electrode materials by observing the SEM images [95].

**Figure 7:**
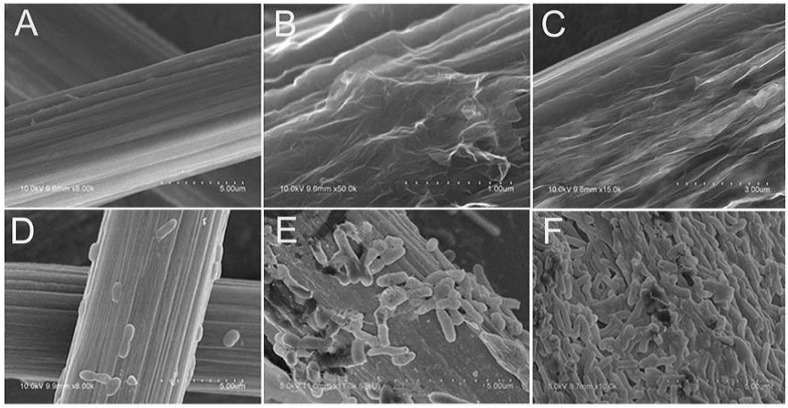
SEM images of carbon paper (CP) (**A**,**D**); graphene nanosheets modified carbon paper (GNS/CP) (**B**,**E**); and graphene nanosheets modified carbon paper with a positively charged ionic liquid (IL-GNS/CP) (**C**,**F**) electrodes before and after *S. oneidensis* cells attached on the surface of the anodes. Adapted from [21], with permission from © 2013 The Royal Society of Chemistry.

Most microorganisms are negatively charged, so Guo et al. [64] prepared graphene-modified carbon paper (CP) electrodes using the electrostatic adsorption reaction between negatively charged graphene and positively charged polyethyleneimine (PEI) with a layer-by-layer method. The electrostatic adsorption reaction greatly increased the number of microorganisms attached on the positively charged graphene-modified carbon paper electrodes. The maximum power density of the MFC based on this type of anode electrodes reached a value of up to 368 mW·m^−2^, which was two times higher than that of the MFC based on unaltered electrodes. Zhao et al. [21] created modified graphene nanosheets (GNS) with a positively charged ionic liquid on CP (IL-GNS/CP) electrode to improve the interactions between the anode electrodes and the microorganisms. The number of microorganisms attached on the IL-GNS/CP electrodes was significantly more than that on the CP and GNS modified CP (GNS/CP) electrodes (Figure 7). The maximum power density of the IL-GNS/CP increased to 601 mW·m^−2^, which is much higher than the value for the control groups. Meanwhile, Han et al. [95] considered a thermal treatment that could introduce some hydrophilic functional groups on the surface of electrodes, for instance, C=N–C and N–C=O, and enhance the biocompatibility. The prominent hierarchical and porous structure of the graphene-modified electrodes makes it possible to enable microorganisms to attach on both the inside and outside surface of the pores [78].

#### 3.1.3. EET Efficiency

Compared to biocompatibility, EET is a process in which the electrons stored in the substrate are released by microorganism metabolism and transferred to the surface of the electrodes [70,96]. There are approximately three extracellular electron transfer mechanisms from microorganisms to electrodes: (1) direct electron transfer based on redox proteins on the bacterial surface, such as outer-membrane c-type cytochrome proteins (*MtrC*, *OmcA*, *OmcB* or *OmcZ*) [70,97]; (2) intercellular “nanowires”, i.e., the electrically conductive pili [55,98]; and (3) indirect electron transfer based on electrochemically active metabolites, such as riboflavin molecules in solution [23,99]. The active sites of outer-membrane c-type cytochromes, covered with non-conductive peptide chains, strongly impede the EET efficiency [100]. As Jain et al. [101] have suggested, the biofilm can be subdivided into three regions: (1) the dense inner core, the electrochemically active inner core that mainly contributes to the EET process; (2) the electron acceptor limitation zone, the intermediate zone that contributes partially to the EET process; and (3) the metabolically inactive zone, the top layer of the biofilms that contributes slightly to this process. In addition, the outer-membrane cytochrome proteins that accumulate on the biofilm surface play an extremely important role in the short-distance EET, and the existence of nanowires is beneficial to enhancing the EET process at long distances [55].

**Figure 8:**
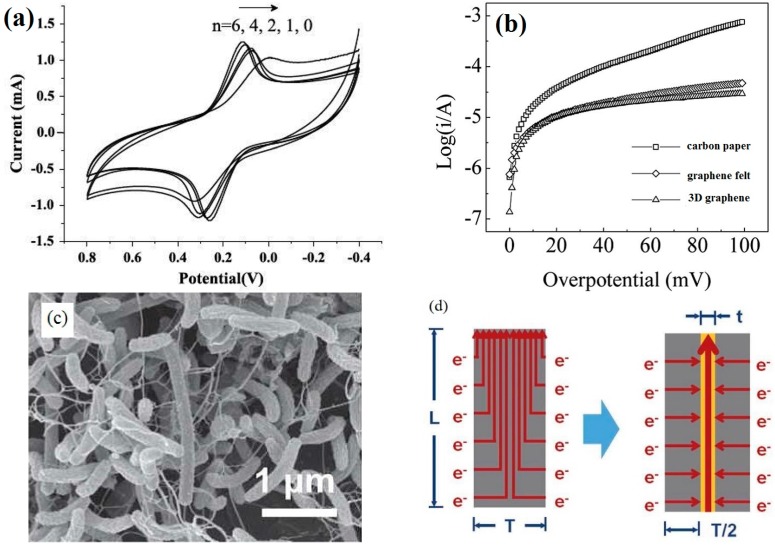
(**a**) Cyclic voltammograms of n-layer graphene composites (n = 0, 1, 2, 4, 6) in aqueous 0.1 mol L^−1^ KCl containing 10 mmol L^−1^ K_3_[Fe(CN)_6_] at a scan rate of 20 mV s^−1^. Adapted from [98], with permission from © 2015 The Royal Society of Chemistry; (**b**) Tafel plots of the different cultured anodes. Adapted from [102], with permission from © 2016 The Royal Society of Chemistry; (**c**) Microorganisms interconnected via microbial nanowires. Adapted from. Adapted from [66], with permission from © 2012 The Royal Society of Chemistry; (**d**) Schematic of the extracellular electron transfer (EET) pathways in the graphene sponge (GS) electrode with (right) and without (left) stainless steel. Adapted from [66], with permission from © 2012 The Royal Society of Chemistry.

As shown in Figure 8a, the oxidative/reductive peak separations (△*E*_p_) of the graphene-modified carbon paper electrodes were smaller than those of the blank carbon paper electrodes, indicating that the existence of graphene in the anode electrodes improved the EET kinetics between the outer-membrane cytochrome proteins and the solid electrodes [98]. The EET kinetics parameters, such as the exchange current density and the transfer coefficient, can be obtained from Tafel plots (Figure 8b) [102]. To reduce the interfacial charge-transfer resistance and enhance the EET efficiency, the traditional carbon based electrodes are always modified with graphene because of the excellent electrical conductivity of grapheme (10^6^ S·m^−1^) [55,103]. The graphene scaffold acted as an enhanced nanowire in the composite electrodes, improving the EET efficiency (Figure 8c). Although graphene has a high electrical conductivity, the GO defects and the gaps between the graphene sheets disrupted the EET efficiency [1]. For some conducting polymers, metal or metallic oxide was applied in the preparation of the electrodes. Zhao et al. [57] fabricated graphene-modified carbon paper coated with a PANI network, which not only significantly increased the active surface area for the immobilization of microorganisms but also enhanced the direct EET via outer-membrane cytochromes. In addition, the pore size of the PANI network was less than the pore size of the microorganisms, thus providing a large surface area for the attachment of microbial secretions and increasing the EET efficiency [57]. Xie et al. [66] inserted stainless steel into two pieces of GS composites. The use of stainless steel reduced the ohmic resistance from 180 Ω to 22 Ω, forming an electron transport “highway” (Figure 8d). The low resistance, including a low charge-transfer resistance and a low ohmic resistance, resulted in the high EET rates [33,54,104].

#### 3.1.4. Mechanical Properties

**Figure 9:**
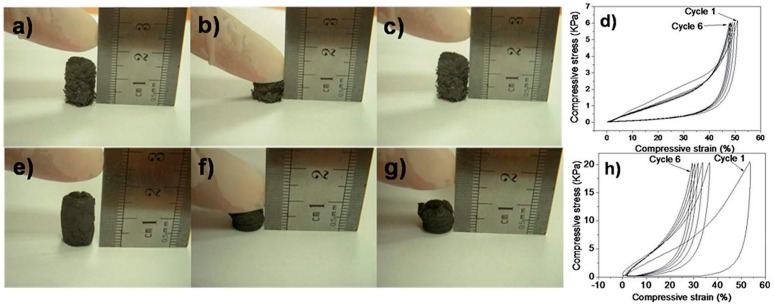
Mechanical properties of graphene sponges (GS) and graphene foams (GF). Compression-recovery process of GS (**a**–**c**) and GF (**e**–**g**) after 50% deformation, and the compressive stress-strain curves of 6 cycles of loading and unloading for GS (**d**) and GF (**h**) under constant pressure. Adapted from [3], with permission from © 2014 The Royal Society of Chemistry.

Although graphene nanosheets exhibit excellent mechanical properties [105,106,107] (the breaking strength and Young’s modulus of defect-free graphene are as high as 42 N·m^−1^ and 1.0 TPa, respectively), the mechanical strength of large-scale graphene is not preserved in electrodes for real applications. To address this situation, a series of measures should be taken. As the electrodes for MFCs, graphene is always modified on the substrate material [55,57,84,97], such as carbon paper, carbon cloth, and a stainless steel mesh, via the dipping-and-drying process [1], spraying [49], electrostatic interaction [21], the explosion method [71], the ammonia-evaporation-induced method [77], and the utilization of a cross-linking agent [108]. In these ways, the electrode materials not only keep the good mechanical properties of substrate materials but also introduce the superior electrical conductivity of graphene. To maintain the porous structure, polyurethane sponge [66], melamine foam [1], and nickel foam [109] are used as templates to form graphene-modified materials. Xie et al. [66] prepared a GS electrode using polyurethane sponge as a template. The graphene layers coated on the surface of the polyurethane sponges were not peeled off after the Scotch-tape test and water flush test (100 mL·min^−1^) were conducted for ten minutes. Meanwhile, Chen et al. [3] controlled the temperature and freezing rate to obtain GA with different pore sizes. GS with a large pore size recovered well when it was compressed into half its volume (Figure 9a–c), and GF recovered partially (Figure 9e–g). The compressive stress-strain curves of six cycles of loading and unloading indicate that GS has more flexibility than GF (Figure 9d,h). The practical application of MFCs becomes possible only once the mechanical properties of a graphene modified electrode material are enhanced.

Many studies suggested that graphene and its composite electrodes enhanced the electricity generation performance of MFCs. The micron-sized pores of graphene-modified electrodes not only prevented the blocking created by the attachment of microorganisms but also improved the biocompatibility and EET efficiency [23,104]. The reasons for the electricity generation performance of the MFCs were as follows: (1) the macroporous structure of the graphene-modified materials increased the active surface area, enhancing the interaction between the multi-biofilms and the electrodes; (2) the macropores provided more surface for the attachment of microorganisms, and the micropores contained the microbial metabolite that improved the EET ability; (3) the graphene architecture worked as the nanowires enhanced the electrical conductivity, reduced the polarization phenomenon and improved the energy exchange efficiency [4,57,68]. Table 1 summarizes the MFC anode electrode studies reported in recent years.

### 3.2. Design and Development of Graphene-Modified Cathode Materials in MFCs

Although the biodegradation process occurred in the MFC anode chamber, the output power depended, to a large extent, on the oxygen reduction reaction (ORR) in the cathode chamber. Platinum (Pt) is always used as the catalyst in the cathode reaction because of its small overpotential, but its high cost has hindered its use in large-scale application MFCs [73]. Thus, it is essential to manufacture high-activity and low-cost cathode materials. In addition, the electricity generation performance of MFCs is affected by electron acceptors, such as oxygen, potassium ferricyanide, and potassium permanganate. Among them, oxygen is deemed to be an ideal electron acceptor at the MFC cathode because it is easily accessible from air. Graphene-modified materials provide a large number of possible active sites and strengthen the reduction pathways.

#### 3.2.1. Reduction Pathways

According to the catalytic ability of the cathode catalysts, ORR always proceeds via a 4-electron pathway with noble metals, such as platinum and palladium (Pd), as the catalysts, or a 2-electron pathway with non-noble metals, such as carbon based materials, as the catalysts [50]. The electrons coming from the anode electrodes can be accepted by the cathode electron acceptors, including nitrate, chromium, and ferricyanide. Among them, O_2_ is the most preferred electron acceptor because of its low cost, ubiquity and high standard potential. There are two different mechanisms to understand how O_2_ is reduced into OH^−^ [111,112]: (1) a direct reaction can proceed between O_2_, electrons and protons via a 4-electron pathway (Equation (1)); (2) an inefficient 2-electron pathway can proceed with ${HO}_{2}^{-}$ as the intermediate product (Equation (2)), followed by the reduction of ${HO}_{2}^{-}$ (Equation (3a)) or a disproportionation reaction between ${HO}_{2}^{-}$ and the solution (Equation (3b)). The hydrogen peroxide production, with a high overpotential, not only increases the energy loss but also damages the membrane separating the anode and cathode chambers [113].

O_2_ + 4H^+^ + 4e^−^ → 2H_2_O, *E^θ^* = 1.229 V vs. SHE (1)

O_2_ + H_2_O + 2e^−^ → HO_2_^−^ + OH^−^, *E^θ^* = −0.076 V vs. SHE (2)

HO_2_^−^ + H_2_O + 2e^−^ → 3OH^−^, *E^θ^* = 0.878 V vs. SHE (3a)

2HO_2_^−^ → 2OH^−^ + O_2_ (3b)

**Figure 10:**
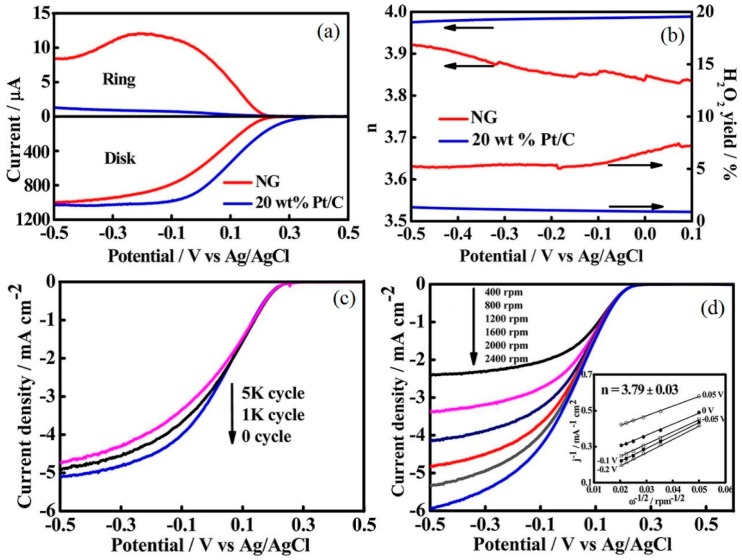
Variation of disk and ring currents on nitrogen-doped graphene (NG) cathode and Pt/C cathode as a function of the potential at a rotation rate of 1600 rpm (**a**); and the corresponding H_2_O_2_ yield and electron transfer number (*n*) as a function of the potential (**b**). Polarization curves (*j*-V relationship, where *j* is represent for the current density) of oxygen reduction reaction (ORR) at NG cathode (**c**) after varying the potential cycles at a rotation rate of 1600 rpm, and (**b**) at varied rotation rates after 5000 cycles, inset (**d**) shows the Koutecky-Levich (k-l) plots. Adapted from [50], with permission from © 2013 American Chemical Society.

The ORR pathway can be explored through cyclic voltammetry curves and polarization curves [50]. The electron transfer number of ORR can be calculated from the slopes of Koutecky-Levich (k-l) plots by the followed equation:

(4) $$\frac{1}{i}=\frac{1}{i_{k}}+\frac{1}{i_{d}}$$

(5) $$i_{d}=0.62nFA\nu^{-1/6}C_{O_{2}}{D_{O_{2}}}^{2/3}\omega^{1/2}$$

where *i* is the measured current density, *i_k_* is the kinetic current density, *i_d_* is the diffusion limiting current density, *ω* is the rotating speed of the disk electrode (rad·s^−1^), n is the number of electrons transferred per oxygen molecule in the ORR process, *F* is the Faraday constant (96,485 C·mol^−1^), *A* is the area of the disk electrode, *D*_O_2__ is the diffusion coefficient of O_2_, *ν* is the kinetic viscosity of the electrolyte, and *C*_O_2__ is the bulk concentration of O_2_ [114,115]. The modification of heteroatoms over the graphene sheets produces asymmetric spin densities, atomic charge densities, and mass defects, which generate a large active area on the surface of graphene sheets [116]. Nitrogen-doped graphene (NG) has shown an excellent electrocatalytic activity and a long-term stability [71]. According to Equations (4) and (5), the electron transferred (*n*) for ORR was calculated to be 3.87 ± 0.04, which is extremely close to the value from the 4-electron transfer pathway of the H_2_O product, indicating that the ORR on NG proceeded via a combined pathway involving 2-electron and 4-electron processes (Figure 10) [50].

In addition, the type of the N species used makes a difference in the nitrogen-doped materials [117]. Graphitic N can reduce O_2_ to H_2_O_2_, via adsorbed HO_2_^−^ as the intermediate through a 2-electron pathway, while pyridinic and pyrrolic N species can reduce the adsorption energy of O_2_, which can convert the ORR mechanism from a 2-electron dominated pathway to a 4-electron pathway [50,118]. Hence, a high content of pyridinic and pyrrolic N may strengthen the ORR mechanism of NG through 4-electron pathways. In considering the high catalytic performance of α-MnO_2_, Khilari et al. [113] demonstrated that graphene-supported α-MnO_2_ had a similar behavior to Pt as a catalyst for the 4-electron pathway of the ORR mechanism, and the performance of MFC-based graphene-supported α-MnO_2_ was comparable to that of the Pt/C electrode in terms of OCV, maximum power density, and COD removal. The low interfacial charge-transfer resistance helped to reduce the overpotential of ORR. Studies suggested that metal, metal oxide, and conducting polymers anchored over the surface of graphene enhanced the ORR activity by increasing the active sites of the graphene nanosheets [45,116,119].

#### 3.2.2. Active Sites

Although Pt exhibited a high catalytic performance as an optimal catalyst, the components of wastewater can damage the stability of Pt cathode electrodes [111]. OH^−^ and Cl^−^ in wastewater were adsorbed on the surface of the cathode electrodes; they occupied the active sites, leading to the disconnection between O_2_ and the electron coming from the anode chambers via the external circuit [120,121]. Due to the pyridinic and pyrrolic N in NG that can reduce the adsorption energy of O_2_, the NG electrodes with a high nitrogen content and a porous structure provided more active sites to facilitate the oxygen reduction, exhibiting a higher electrocatalytic activity towards ORR [79,122]. The interlayer spacing between the graphene nanosheets can be expanded in the redox reaction, which provided a much larger catalyst surface area and increased the catalytically active sites. In addition, the macro and mesopore structure of the graphene material were beneficial for oxygen transfer, which made it easy for oxygen to reach the catalytically active sites, leading to a decrease in the diffusion resistance; the micropores also provided additional active sites for ORR [79,111,123].

**Figure 11:**
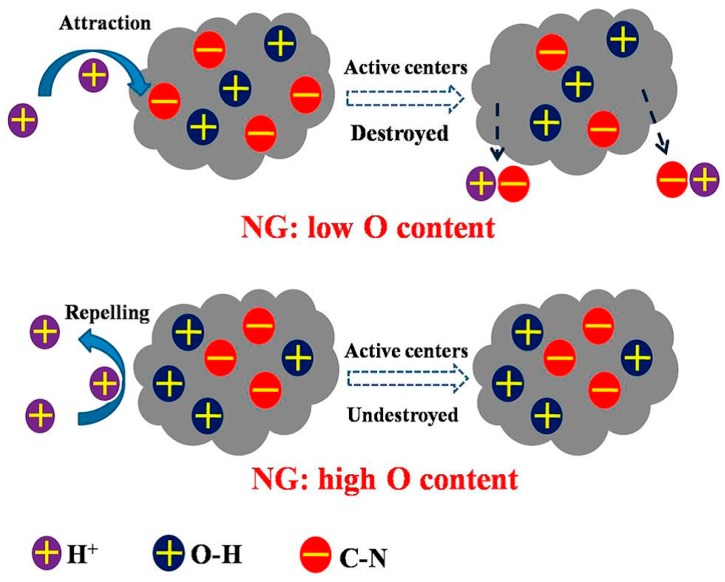
C-N groups on the surface of nitrogen-doped graphene (NG) were protected from the assault of protons by O-H groups. Adapted from [50], with permission from © 2013 American Chemical Society.

Furthermore, the O species can also impact the long-time stability of NG in a neutral pH medium. The negatively charged C-N groups that behaved as active centers for ORR can be destroyed in the long run, which resulted in the decline of the active sites. However, the high content of positively charged O-H groups can potentially improve the proton-tolerance ability during long operation times [50,55]. Hence, the O-H groups can prevent the C-N groups from being destroyed (Figure 11). The functional groups that act as conductive active sites on the surface of electrodes, such as C≡N, C=O, O-C and C-O-C, can decrease the activation energy barrier by reducing the electrode double-layer thickness [124]. Metal, metal oxide, and conductive polymers modified the graphene material and improved the number of active sites [116]. However, some studies shown that the Fe element can only promote the formation of active centers, but it cannot function as an active center site [76,125]. In addition, the large surface area of graphene materials can provide additional active sites, which can enhance the ORR catalysis [111]. In the operation of MFCs, the catalysts may lose their active sites via a severe surface oxidation reaction, leading to the corrosion of the electrode surface.

#### 3.2.3. Electrical Conductivity

Compared with the anode electrode, there are no microorganisms attached on the surface of the cathode electrode, except for the MFCs that use aerobic microorganisms as electron acceptors. Therefore, the preferred method for enhancing the electrical conductivity is to improve the surface modification of the electrodes. Although pure graphene nanosheets have excellent electrical conductivity [103] (10^6^ S·m^−1^), the defects and functional groups on their surface can decrease the electrical conductivity [19], which limits the overall performance of MFCs. Electron transfer should be conducted through the triple phase boundary reaction [126]. A high electrical conductivity shows that the overall inner resistance of the MFC decreased, assisting an effective electron transfer between the electrodes and electron acceptors [116]. To reduce the cathodic ohmic loss, effective ORR catalysts should be modified on cathode materials such as SnO_2_, Ag, PANI, and the P element [53,127,128,129,130]. The electrical conductivity can be measured by using a four-point probe system [49,131]. Nitrogen-doped graphene materials exhibited excellent electrical conductivity in the bulk graphene materials, and the electrical conductivity of NG cathodes was 35 ± 3 S·cm^−1^, which was 10 S·cm^−1^ lower than the conductivity of cathodes based on Pt/C [71]. The NG catalyst showed a much better electrocatalytic activity and improved long-term operation stability for the ORR process than the Pt catalyst [119]. Similar to the N element, the electronegativity of the P element is less than C, which can enhance the positive charge density, resulting in more active sites for the adsorption of oxygen [130].

Conductive polymers, such as polypyrrole (PPy) and PANI, possess an extended π conjugation along their backbone, indicating their high electrical conductivity and electron affinities [111,116]. Ren et al. [51] doped graphene into PANI to ameliorate the cathode performance. Although MnO_2_ has a considerable catalytic activity for the electrochemical ORR, the poor electrical conductivity of MnO_2_ (10^−4^–10^−5^·S·cm^−1^) limits its application in MFCs [49,53,113]. The strong interaction between MnO_2_ and graphene material improved the electrical conductivity to 0.09 S·cm^−1^, and the maximum power density of the MFC based on this cathode materials increased to 3359 mW·m^−2^, which was much higher than that based on the traditional Pt/C catalyst [49]. In addition, to reduce the overall resistivity of MFCs, multiple factors, such as the electrode conductivity for both the anode and cathode electrode), electrolyte conductivity, membrane conductivity and mass transport, should be improved [132]. Furthermore, the electrode distance and the electrode overpotential should also be reduced; the use of electron acceptors and electrolyte acidification should also be taken seriously.

#### 3.2.4. Electron Acceptor

**Figure 12:**
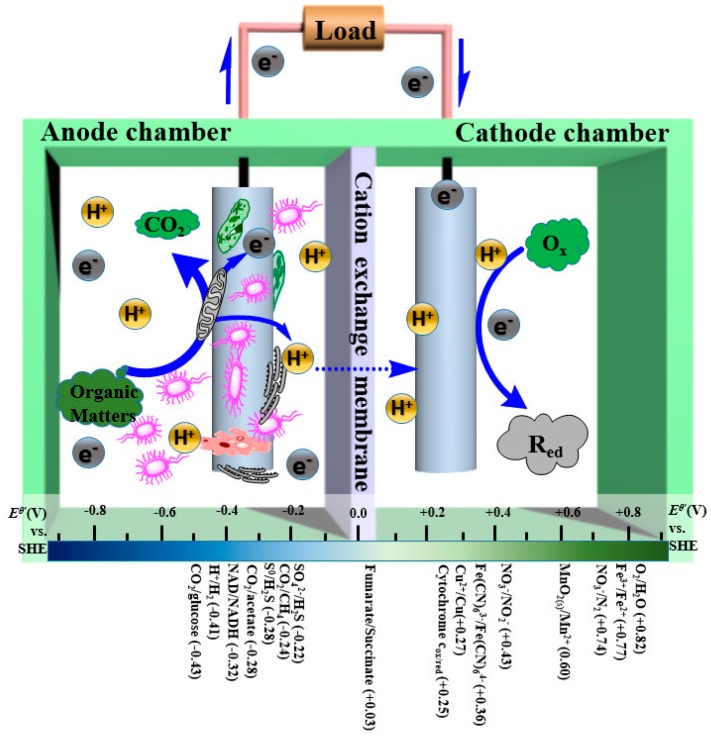
Fundamental configuration of microbial fuel cells with the redox potential of various electron acceptors and donors at pH = 7.0.

The electron acceptor is considered to be one of the most important factors in overcoming the potential losses in the cathode chamber. Electron acceptors can be divided into inorganic electron acceptors (oxygen, nitrogen-containing compounds, and metal-containing ions) and organic electron acceptors (azo dyes, nitrogenous aromatic compounds, and chlorophenols) [133,134]. Figure 12 shows the redox potential (vs. standard hydrogen electrode) of various electron acceptors. Among them, oxygen is known as the most sustainable and suitable electron acceptor due to its low cost, high redox potential, and availability in the environment. Therefore, the air-cathode single-chamber MFC is commonly used to utilize the oxygen in air [18,135]. However, the leakage of oxygen from the cathode chamber to the anode can poison the anaerobic bacteria in the anode chamber [20]. To use the oxygen in the air directly rather than through aeration, the air cathode is the best choice; it is manufactured by pressing wet-proof gas diffusion layers, the catalyst layer, and the cathode electrode [79].

To reduce the overpotential of electron acceptors and maintain a steady cathode potential, Yong et al. [70] filled the cathode chamber with 0.5 mM K_3_[Fe(CN)_6_] to study the effect of polyaniline-hybridized 3D graphene on the anode performance. There are two problems to be faced when these nitrogen-containing compounds and metal-containing ions are used as electron acceptors: (1) the catholyte has to be exchanged regularly, which is a waste of resources; (2) the exhausted catholyte can act as a new contaminant. The cathode chamber can be inoculated with aerobic activated sludge [136]. Studies indicated that the maximum power density of MFCs based on the graphene-biocathode increased by 103% compared with the MFCs based on the carbon cloth biocathode, and the electron acceptor of these MFCs was oxygen supplied to the cathode chamber with an air pump [60]. Because the MFC based on biocathodes can function with a high efficiency, a new-style microalgae MFC has appeared; it has the capacity to convert solar energy into electricity via the metabolism of photosynthetic microorganisms [137]. The use of membrane-less MFCs based on biocathodes shows a remarkable achievement with a 24 h hydraulic retention time, in which the rate of the removal of COD and ${NH}_{4}^{+}$ approached 90% and 99%, respectively [138]. In the future, aerobic biocathodes can utilize inorganic compounds, such as nitrate, sulfate, and iron, in wastewater as terminal electron acceptors [139], which provides a new way of degrading inorganic salt.

Although degradation of organic matter occurred in the anode chamber, the electron acceptor is in the cathode chamber, and the electron acceptability and consumption ability can affect the oxidation rate in the chamber to a large extent. Hence, it is essential for the cathode electrode to have a high electron transfer capability and fast ORR rate [51]. Due to the outstanding electrical conductivity and high specific surface area of graphene and its compounds, the active sites of the cathode materials are sharply increased, which results from the increased mutual contact between the catalysts and electrolyte, indicating that a high electron transfer efficiency and a low inner resistance. Table 2 shows the summary of studies on the MFC cathode electrodes reported in recent years.

## 4. Conclusions and Outlook

Although a series of challenges faces the practical application of MFCs, the development of MFCs for wastewater treatment has concerned many researchers for a long time. There has been undeniably great progress in enhancing the performance of MFCs, and various reactor configurations have been designed to explore the operating principle of MFCs, e.g., H-shaped MFCs [78], air-cathode single-chamber MFCs [20,79] and single-chamber membrane-free MFCs [18]. The reactions all occur on the surface of electrode materials, whether the microorganism catalytic degradation of organic matter in the anode chamber or the ORR of the electron acceptor in the cathode chamber. Hence, the excellent properties of electrode materials are the essential factor in determining the electricity generation performance of MFCs, which is significant for their practical application. Due to the excellent physical, chemical, and biological performance of graphene and its compounds, as mentioned earlier, they have gradually become one of the most popular materials in MFC research. In this review, we have shown that 3D porous graphene-based materials have a higher specific surface area, an improved electrical conductivity, and a more outstanding catalytic performance than traditional materials such as carbon paper, carbon cloth, and graphite particles, which indicates that graphene and its compounds are the ideal electrode materials in MFCs. With the use of modified graphene in the anode chamber, large defective sites are produced on these materials, enhancing their catalytic performance and electron transfer ability and reducing the polarization phenomenon. In the cathode chamber, the superior active sites and the hydrophilicity of graphene-modified materials are beneficial for the interconnection between the catalyst and the electrolyte; this interconnection can improve the ORR rate. The major areas for future studies are to develop the affordability, superior electrical conductivity, high catalytic activity, and outstanding biocompatibility of 3D graphene materials.

In the operation of MFCs, multiple purified exoelectrogens were separated from anaerobic sludge, such as *Escherichia coli*, *Pseudomonas aeruginosa*, and *Shewanella oneidensis* MR-1 leading to the situation that the mechanism of action among these purified exoelectrogens was not clarified (i.e., whether they act by synergistic effect or inhibiting effect), which is important to enhance the performance of MFCs. Partial exoelectrogens have a good electricity generation ability in alkaline condition that enables their use in some conductive compounds, such as PANI, which could then be developed as a new technology to address the alkaline industrial wastewater. In addition, the use of expensive membranes, such as Nafion 117, severely limits the practical application of some MFCs. Therefore, developing a membrane-free MFC or seeking alternative mediator materials, such as salt bridges, instead of these costly membranes could significantly reduce the construction cost of MFCs. To improve the utilization efficiency of oxygen, the cathode can be designed as a biodegradation reactor, such as an aerobic biological reactor in sewage treatment plants, which can effectively accept the electrons coming from the anode chamber while degrading contaminants in the wastewater.

The study of MFCs is an interlaced subject, built on the basis of physics, chemistry, and biology. MFCs can be combined with other technologies, such as membrane bioreactor (MBR) technology that can not only enhance the electrical generation performance but also increase the removal rate of contaminants [145]. In addition, power management systems (PMS) that harvest energy are crucial for the scale-up and practical application of MFCs [146]. However, there are many factors that impact the electrical generation performance of MFCs, for instance, the external resistance, nutrient solution system, microorganism species, and electrode materials. Hence, it is difficult to compare systems to determine which one is better than the others. With the further development of graphene-modified MFCs, a steady operating MFC system can be developed to evaluate the performance of MFCs. Overall, graphene and its compounds, as the ideal electrode materials, increase the performance of MFCs, and they can serve as the core technology needed to address organic wastewater in the future.

## Figures and Tables

**Table 1:** Summary of microbial fuel cells (MFC) anode electrode studies.

No.	S/D	Anode			Membranes	Inoculation	Cathode Electrode	*R_E_*/Ω	OCV/mV	Power Density/mW·m^−2^	Ref.
		Electrode	Modification	Volume/mL							
1	D	3D GF	PANI	150	Nafion 117	*S. oneidensis* MR-1	Carbon cloth	2000	>250	768	[70]
2	D	Graphite felt	PPy/GO	25	CEM	*S. oneidensis* MR-1	Carbon felt	500	~350	1326	[56]
3	D	Carbon paper	GNRs/PANI	100	Nafion 211	*S. oneidensis* MR-1	Carbon paper	1000	-	856	[57]
4	D	Carbon paper	Graphene/Au	100	Nafion 211	*S. oneidensis* MR-1	Carbon paper	1000	-	508	[97]
5	D	Nickel foams	Graphene/TiO_2_	100	Nafion 112	*S. oneidensis* MR-1	Carbon paper	-	-	1060	[22]
6	D	Carbon cloth	Graphene	180	Nafion 117	*P. aeruginosa*	Carbon cloth	1960	~700	52.5	[55]
7	D	Carbon cloth	rGO-SnO_2_	75	Nafion 117	*Escherichia coli*	Pt rod	550	830	1624	[67]
8	D	Ni foam	rGO	25	-	*S. oneidensis* MR-1	-	100	620	661 W·m^−3^	[109]
9	D	Carbon cloth	NGNS	10	Nafion 115	*Escherichia coli*	Carbon cloth	510	~350	1008	[2]
11	S	Glassy carbon	Microbially reduced graphene	10	CEM	Anaerobic activated sludge	Carbon cloth/Pt	1000	>450	1905	[23]
13	S	Graphite block	Graphene	100	-	*S. oneidensis* MR-1	Carbon paper	500	150	102	[54]
14	D	Carbon cloth	PANI-rGO	40	Nafion 117	anaerobic sludge	Carbon felts	500	770	1390	[41]
15	D	Carbon cloth	TiO_2_/rGO	100	Nafion 117	*S. putrefaciens* CN32	Carbon fiber brush	1500	-	3169	[58]
16	D	CHI/VSG scaffolds			Nafion 117	*P. aeruginosa*	Carbon cloth	1960	910	1530	[68]
17	D	Carbon cloth	Graphene	20	NO	*Escherichia coli* B	Carbon cloth	-	900	2850	[110]
18	S		3D-Graphene	28	-	Previous reactor	Carbon cloth/Pt	1000	-	1516 ± 87	[102]
19	D	3D GS aerogels		120	CMI7000	Anaerobic sludge	Carbon paper	1000	~550	710	[3]
20	D	Carbon cloth	GA	100	Nafion 117	*S. putrefaciens* CN32	Carbon cloth	1500	~700	679.7	[4]
21	S	Carbon paper	Graphene with IL-NH_2_		-	*S. oneidensis* MR-1	-	-	-	610	[21]
22	D	Stainless-steel mesh	Graphene-containing foam	-	Nafion 117	*S. putrefaciens*	Carbon paper	-	~600	768	[33]
23	D	Carbon cloth	rGO/PPy	-	-	*Escherichia coli*	-	1000	400	1068	[42]
24	D	Carbon paper	Graphene	140	CMI-7000	Anaerobic sludge	Carbon paper	1000	580	368	[64]
25	D	Polyurethane	GS	-	-	Previous reactor	Carbon cloth/Pt	475	-	1570	[66]
26	D	Stainless-steel mesh	Graphene	115	Nafion 112	*Escherichia coli*	Carbon paper	-	790	2668	[84]
27	S	Ni foam	3D rGO	20	-	*Escherichia coli* K12	Carbon caper/Pt	1000	623	897.1	[85]

In the second column, “S” is for the single-air cathode MFC and “D” is for the dual-chamber MFC including the “H style” MFC. “-” means that it is not mentioned in the research. In the fourth column, “NO” represents membrane-free, and CEM represents cation exchange membrane. *R_E_* means the external resistance.

**Table 2:** Summary of MFC cathode electrode studies.

No.	S/D	Cathode		Membranes	Inoculation	Anode		External Resistor/Ω	OCV/mV	Power Density/mW·m^−2^	Ref.
		Electrode	Modification			Electrode	Volume/mL				
1	D	Carbon paper	NG	Nafion 117	Activated sludge	Carbon cloth	100	1000	650	776 ± 12	[50]
2	D	Glassy carbon	Fe- and N-functionalized graphene	Nafion 117	Previous reactor	Carbon felt	~30	1000	570	885	[76]
3	S	Carbon paper	MnO_2_-NTs/graphene	Nafion 117	Previous reactor	Carbon cloth	60	1000	613	4.68 W·m^−3^	[113]
4	S	Carbon cloth	Pt-Co/G	NO	Previous reactor	Carbon cloth	27	1000	710	1378	[135]
5	D	Carbon cloth	Graphene/biofilm	Nafion 117	Anaerobic activated sludge	Carbon cloth	10	1000	390	323.2 ± 21	[60]
6	D	Carbon cloth	NG	NO	Previous reactor	Carbon fiber brush	20	1000	555	1350 ± 15	[71]
7	S	Stainless steel net	NG	-	Anaerobic sludge	Carbon brush	200	1500	243	1159.34	[79]
8	S	Carbon Paper	Fe-NG	CEM	Anaerobic activated sludge	Carbon felt	40	500	242	1149.8	[119]
9	S	Stainless steel net	MnO_2_/GNS	-	Anaerobic sludge	Carbon felt	200	1500	771	2083	[53]
10	S	Carbon cloth	α-MnO_2_/GO	Nafion 117	Sewage sludge	Carbon cloth	25	-	710	3359	[49]
11	S	Stainless steel mesh	Cobalt sulfides/GO	NO	Previous reactor	Graphite fiber	28	1000	620	1156 ± 18	[140]
12	D	Carbon cloth	NG/CoNi-alloy	CEM	-	Carbon brush	140	1000	~700	2000	[141]
13	S	PANI	Graphene	NO	Residual sludge	Graphite	1800	500	640	99	[51]
14	D	Carbon cloth	rGO particles	Ultex CMI 7000	Anaerobic sludge	Carbon brush	120	1000	650	3.3 W·m^−3^	[52]
15	D	Carbon paper	Graphene with Iron tetrasulfophthalocyanine.	Nafion 112	*Escherichia coli*	Carbon paper	115	-	-	817	[45]
16	S	Carbon cloth	Graphene/Pt	NO	*Escherichia coli*	Carbon cloth	75	1000	~260	0.159	[18]
17	D	Carbon cloth	NG	-	-	Carbon fiber brush	120	-	840	4.06 W·m^−3^	[142]
18	D	Graphene-Au-laccase hybrid		PFSA NRE-211	*Trametes versicolor*	Graphene-Au	-	-	1160	1.96 mW·cm^−2^	[6]
19	S	Carbon paper	Graphene/PANI	-	-	-	-	-	593	17.95	[143]
20	D	Graphite rods	Prussian blue/graphene	Nafion 117	Previous reactor	Graphite rods	80	1000	530	15.63 W·m^−3^	[144]

In the second column, “S” is for the single-air cathode MFC and “D” is for the dual-chamber MFC including the “H style” MFC. “-” means that it is not mentioned in the research. In the fourth column, “NO” represents membrane-free, and CEM represents cation exchange membrane.

## References

[1] Chou H.T., Lee H.J., Lee C.Y., Tai N.H., Chang H.Y. (2014). Highly durable anodes of microbial fuel cells using a reduced graphene oxide/carbon nanotube-coated scaffold. Bioresour. Technol..

[2] Kirubaharan C.J., Santhakumar K., Kumar G.G., Senthilkumar N., Jang J.H. (2015). Nitrogen doped graphene sheets as metal free anode catalysts for the high performance microbial fuel cells. Int. J. Hydrogen Energy.

[3] Chen W.F., Huang Y.X., Li D.B., Yu H.Q., Yan L.F. (2014). Preparation of a macroporous flexible three dimensional graphene sponge using an ice-template as the anode material for microbial fuel cells. RSC Adv..

[4] Qiao Y., Wen G.-Y., Wu X.-S., Zou L. (2015). l-cysteine tailored porous graphene aerogel for enhanced power generation in microbial fuel cells. RSC Adv..

[5] Quan X.C., Mei Y., Xu H.D., Sun B., Zhang X. (2015). Optimization of Pt-Pd alloy catalyst and supporting materials for oxygen reduction in air-cathode microbial fuel cells. Electrochim. Acta.

[6] Chen Y., Gai P.P., Zhang J.R., Zhu J.J. (2015). Design of an enzymatic biofuel cell with large power output. J. Mater. Chem. A.

[7] Mink J.E., Qaisi R.M., Hussain M.M. (2013). Graphene-based flexible micrometer-sized microbial fuel cell. Energy Technol..

[8] Zhang Q., Xu X., Li H., Xiong G., Hu H., Fisher T.S. (2015). Mechanically robust honeycomb graphene aerogel multifunctional polymer composites. Carbon.

[9] Angosto J.M., Fernandez-Lopez J.A., Godinez C. (2015). Brewery and liquid manure wastewaters as potential feedstocks for microbial fuel cells: A performance study. Environ. Sci. Technol..

[10] Dunaj S.J., Vallino J.J., Hines M.E., Gay M., Kobyljanec C., Rooney-Varga J.N. (2012). Relationships between soil organic matter, nutrients, bacterial community structure, and the performance of microbial fuel cells. Environ. Sci. Technol..

[11] Inoue K., Ito T., Kawano Y., Iguchi A., Miyahara M., Suzuki Y., Watanabe K. (2013). Electricity generation from cattle manure slurry by cassette-electrode microbial fuel cells. J. Biosci. Bioeng..

[12] Nimje V.R., Chen C.Y., Chen H.R., Chen C.C., Huang Y.M., Tseng M.J., Cheng K.C., Chang Y.F. (2012). Comparative bioelectricity production from various wastewaters in microbial fuel cells using mixed cultures and a pure strain of *Shewanella oneidensis*. Bioresour. Technol..

[13] Ieropoulos I.A., Ledezma P., Stinchcombe A., Papaharalabos G., Melhuish C., Greenman J. (2013). Waste to real energy: The first MFC powered mobile phone. Phys. Chem. Chem. Phys..

[14] Ieropoulos I.A., Stinchcombe A., Gajda I., Forbes S., Merino-Jimenez I., Pasternak G., Sanchez-Herranz D., Greenman J. (2016). Pee power urinal-microbial fuel cell technology field trials in the context of sanitation. Environ Sci. Water Res. Technol..

[15] Kim K.Y., Chae K.J., Choi M.J., Ajayi F.F., Jang A., Kim C.W., Kim I.S. (2011). Enhanced coulombic efficiency in glucose-fed microbial fuel cells by reducing metabolite electron losses using dual-anode electrodes. Bioresour. Technol..

[16] Kim K.Y., Yang W., Logan B.E. (2015). Impact of electrode configurations on retention time and domestic wastewater treatment efficiency using microbial fuel cells. Water Res..

[17] Kim Y., Hatzell M.C., Hutchinson A.J., Logan B.E. (2011). Capturing power at higher voltages from arrays of microbial fuel cells without voltage reversal. Energy Environ. Sci..

[18] Tsai H.-Y., Hsu W.-H., Huang Y.-C. (2015). Characterization of carbon nanotube/craphene on carbon cloth as an electrode for air-cathode microbial fuel cells. J. Nanomater..

[19] Song T.S., Wang D.B., Wang H.Q., Li X.X., Liang Y.Y., Xie J.J. (2015). Cobalt oxide/nanocarbon hybrid materials as alternative cathode catalyst for oxygen reduction in microbial fuel cell. Int. J. Hydrogen Energy.

[20] Khilari S., Pandit S., Ghangrekar M.M., Pradhan D., Das D. (2013). Graphene oxide-impregnated PVA-STA composite polymer electrolyte membrane separator for power generation in a single-chambered microbial fuel cell. Ind. Eng. Chem. Res..

[21] Zhao C., Wang Y., Shi F., Zhang J., Zhu J.J. (2013). High biocurrent generation in *Shewanella*-inoculated microbial fuel cells using ionic liquid functionalized graphene nanosheets as an anode. Chem. Commun..

[22] Zhao C.E., Wang W.J., Sun D., Wang X., Zhang J.R., Zhu J.J. (2014). Nanostructured graphene/TiO_2_ hybrids as high-performance anodes for microbial fuel cells. Chem. Eur. J..

[23] Yuan Y., Zhou S., Zhao B., Zhuang L., Wang Y. (2012). Microbially-reduced graphene scaffolds to facilitate extracellular electron transfer in microbial fuel cells. Bioresour. Technol..

[24] Krishnamurthy A., Gadhamshetty V., Mukherjee R., Chen Z., Ren W., Cheng H.M., Koratkar N. (2013). Passivation of microbial corrosion using a graphene coating. Carbon.

[25] He Y.R., Xiao X., Li W.W., Sheng G.P., Yan F.F., Yu H.Q., Yuan H., Wu L.J. (2012). Enhanced electricity production from microbial fuel cells with plasma-modified carbon paper anode. Phys. Chem. Chem. Phys..

[26] Baranitharan E., Khan M.R., Prasad D.M.R., Bin Salihon J. (2013). Bioelectricity Generation from palm oil mill effluent in microbial fuel cell using polacrylonitrile carbon felt as electrode. Water Air Soil Pollut..

[27] Chen S.L., He G.H., Carmona-Martinez A.A., Agarwal S., Greiner A., Hou H.Q., Schroder U. (2011). Electrospun carbon fiber mat with layered architecture for anode in microbial fuel cells. Electrochem. Commun..

[28] Taskan E., Hasar H. (2015). Comprehensive comparison of a new tin-coated copper mesh and a graphite plate electrode as an anode material in microbial fuel cell. Appl. Biochem. Biotechnol..

[29] Liu Y., Dustin Lee J.H., Xia Q., Ma Y., Yu Y., Lanry Yung L.Y., Xie J., Ong C.N., Vecitis C.D., Zhou Z. (2014). A graphene-based electrochemical filter for water purification. J. Mater. Chem. A.

[30] Zhuang Y., Yu F., Ma J., Chen J. (2015). Graphene as a template and structural scaffold for the synthesis of a 3D porous bio-adsorbent to remove antibiotics from water. RSC Adv..

[31] Scott S.M., Hu T., Yao T.K., Xin G.Q., Lian J. (2015). Graphene-based sorbents for iodine-129 capture and sequestration. Carbon.

[32] Yang H., Gong J., Wen X., Xue J., Chen Q., Jiang Z., Tian N., Tang T. (2015). Effect of carbon black on improving thermal stability, flame retardancy and electrical conductivity of polypropylene/carbon fiber composites. Compos. Sci. Technol..

[33] Yang L., Wang S., Peng S., Jiang H., Zhang Y., Deng W., Tan Y., Ma M., Xie Q. (2015). Facile fabrication of graphene-containing foam as a high-performance anode for microbial fuel cells. Chem. Eur. J..

[34] Chen W., Li S., Chen C., Yan L. (2011). Self-Assembly and Embedding of Nanoparticles by in situ reduced graphene for preparation of a 3D graphene/nanoparticle aerogel. Adv. Mater..

[35] Chen W., Yan L. (2011). In situ self-assembly of mild chemical reduction graphene for three-dimensional architectures. Nanoscale.

[36] Xu Y., Sheng K., Li C., Shi G. (2010). Self-assembled graphene hydrogel via a one-step hydrothermal process. ACS Nano.

[37] Dong X.-C., Xu H., Wang X.-W., Huang Y.-X., Chan-Park M.B., Zhang H., Wang L.-H., Huang W., Chen P. (2012). 3D graphene-cobalt oxide electrode for high-performance supercapacitor and enzymeless glucose detection. ACS Nano.

[38] Zhang K., Ding C.M., Liu H., Zhu Y., Jiang L. (2014). *Shewanella*-mediated synthesis of reduced graphene oxide film for enhanced extracellular electron transfer. Chem. J. Chin. Univ..

[39] Bi H., Xie X., Yin K., Zhou Y., Wan S., He L., Xu F., Banhart F., Sun L., Ruoff R.S. (2012). Spongy graphene as a highly efficient and recyclable sorbent for oils and organic solvents. Adv. Funct. Mater..

[40] Novoselov K.S., Geim A.K., Morozov S.V., Jiang D., Zhang Y., Dubonos S.V., Grigorieva I.V., Firsov A.A. (2004). Electric field effect in atomically thin carbon films. Science.

[41] Hou J.X., Liu Z.L., Zhang P.Y. (2013). A new method for fabrication of graphene/polyaniline nanocomplex modified microbial fuel cell anodes. J. Power Sources.

[42] Kumar G.G., Kirubaharan C.J., Udhayakumar S., Ramachandran K., Karthikeyan C., Renganathan R., Nahrn K.S. (2014). Synthesis, structural, and morphological characterizations of reduced graphene oxide-supported polypyrrole anode catalysts for improved microbial fuel cell performances. ACS Sustain. Chem. Eng..

[43] Liu Y., Yu L., Ong C.N., Xie J. (2016). Nitrogen-doped graphene nanosheets as reactive water purification membranes. Nano Res..

[44] Fang X.Y., Yu X.X., Zheng H.M., Jin H.B., Wang L., Cao M.S. (2015). Temperature- and thickness-dependent electrical conductivity of few-layer graphene and graphene nanosheets. Phys. Lett. A.

[45] Zhang Y., Mo G., Li X., Ye J. (2012). Iron tetrasulfophthalocyanine functionalized graphene as a platinum-free cathodic catalyst for efficient oxygen reduction in microbial fuel cells. J. Power Sources.

[46] Mao S., Lu G., Chen J. (2015). Three-dimensional graphene-based composites for energy applications. Nanoscale.

[47] Xu L., Zhang G.Q., Chen J., Zhou Y.F., Yuan G.E., Yang F.L. (2013). Spontaneous redox synthesis of Prussian blue/graphene nanocomposite as a non-precious metal catalyst for efficient four-electron oxygen reduction in acidic medium. J. Power Sources.

[48] Li D., Muller M.B., Gilje S., Kaner R.B., Wallace G.G. (2008). Processable aqueous dispersions of graphene nanosheets. Nat. Nanotechnol..

[49] Gnana Kumar G., Awan Z., Suk Nahm K., Xavier J.S. (2014). Nanotubular MnO_2_/graphene oxide composites for the application of open air-breathing cathode microbial fuel cells. Biosens. Bioelectron..

[50] Liu Y., Liu H., Wang C., Hou S.X., Yang N. (2013). Sustainable energy recovery in wastewater treatment by microbial fuel cells: Stable power generation with nitrogen-doped graphene cathode. Environ. Sci. Technol..

[51] Ren Y., Pan D., Li X., Fu F., Zhao Y., Wang X. (2013). Effect of polyaniline-graphene nanosheets modified cathode on the performance of sediment microbial fuel cell. J. Chem. Technol. Biotechnol..

[52] Xiao L., Damien J., Luo J., Jang H.D., Huang J., He Z. (2012). Crumpled graphene particles for microbial fuel cell electrodes. J. Power Sources.

[53] Wen Q., Wang S., Yan J., Cong L., Pan Z., Ren Y., Fan Z. (2012). MnO_2_–graphene hybrid as an alternative cathodic catalyst to platinum in microbial fuel cells. J. Power Sources.

[54] Chen J., Deng F., Hu Y., Sun J., Yang Y. (2015). Antibacterial activity of graphene-modified anode on *Shewanella oneidensis* MR-1 biofilm in microbial fuel cell. J. Power Sources.

[55] Liu J., Qiao Y., Guo C.X., Lim S., Song H., Li C.M. (2012). Graphene/carbon cloth anode for high-performance mediatorless microbial fuel cells. Bioresour. Technol..

[56] Lv Z., Chen Y., Wei H., Li F., Hu Y., Wei C., Feng C. (2013). One-step electrosynthesis of polypyrrole/graphene oxide composites for microbial fuel cell application. Electrochim. Acta.

[57] Zhao C., Gai P., Liu C., Wang X., Xu H., Zhang J., Zhu J.-J. (2013). Polyaniline networks grown on graphene nanoribbons-coated carbon paper with a synergistic effect for high-performance microbial fuel cells. J. Mater. Chem. A.

[58] Zou L., Qiao Y., Wu X.-S., Ma C.-X., Li X., Li C.M. (2015). Synergistic effect of titanium dioxide nanocrystal/reduced graphene oxide hybrid on enhancement of microbial electrocatalysis. J. Power Sources.

[59] Li R., Chen C.B., Li J., Xu L.M., Xiao G.Y., Yan D.Y. (2014). A facile approach to superhydrophobic and superoleophilic graphene/polymer aerogels. J. Mater. Chem. A.

[60] Zhuang L., Yuan Y., Yang G., Zhou S. (2012). In situ formation of graphene/biofilm composites for enhanced oxygen reduction in biocathode microbial fuel cells. Electrochem. Commun..

[61] Erbay C., Yang G., de Figueiredo P., Sadr R., Yu C.H., Han A. (2015). Three-dimensional porous carbon nanotube sponges for high-performance anodes of microbial fuel cells. J. Power Sources.

[62] Ma J., Yang M., Yu F., Zheng J. (2015). Water-enhanced removal of ciprofloxacin from water by porous graphene hydrogel. Sci. Rep..

[63] Xu Y.X., Lin Z.Y., Huang X.Q., Wang Y., Huang Y., Duan X.F. (2013). Functionalized graphene hydrogel-based high-performance supercapacitors. Adv. Mater..

[64] Guo W., Cui Y.R., Song H., Sun J.H. (2014). Layer-by-layer construction of graphene-based microbial fuel cell for improved power generation and methyl orange removal. Bioproc. Biosyst. Eng..

[65] Sun J.-J., Zhao H.-Z., Yang Q.-Z., Song J., Xue A. (2010). A novel layer-by-layer self-assembled carbon nanotube-based anode: Preparation, characterization, and application in microbial fuel cell. Electrochim. Acta.

[66] Xie X., Yu G.H., Liu N., Bao Z.N., Criddle C.S., Cui Y. (2012). Graphene-sponges as high-performance low-cost anodes for microbial fuel cells. Energy Environ. Sci..

[67] Mehdinia A., Ziaei E., Jabbari A. (2014). Facile microwave-assisted synthesized reduced graphene oxide/tin oxide nanocomposite and using as anode material of microbial fuel cell to improve power generation. Int. J. Hydrogen Energy.

[68] He Z.M., Liu J., Qiao Y., Li C.M., Tan T.T.Y. (2012). Architecture engineering of hierarchically porous chitosan/vacuum-stripped graphene scaffold as bioanode for high performance microbial fuel cell. Nano Lett..

[69] Kim K.S., Zhao Y., Jang H., Lee S.Y., Kim J.M., Kim K.S., Ahn J.H., Kim P., Choi J.Y., Hong B.H. (2009). Large-scale pattern growth of graphene films for stretchable transparent electrodes. Nature.

[70] Yong Y.C., Dong X.C., Chan-Park M.B., Song H., Chen P. (2012). Macroporous and monolithic anode based on polyaniline hybridized three-dimensional graphene for high-performance microbial fuel cells. ACS Nano.

[71] Feng L., Chen Y., Chen L. (2011). Easy-to-operate and low-temperature synthesis of gram-scale nitrogen-doped graphene and its application as cathode catalyst in microbial fuel cells. ACS Nano.

[72] Ambrosi A., Bonanni A., Sofer Z., Pumera M. (2013). Large-scale quantification of CVD graphene surface coverage. Nanoscale.

[73] Zhou L.H., Fu P., Wen D.H., Yuan Y., Zhou S.G. (2016). Self-constructed carbon nanoparticles-coated porous biocarbon from plant moss as advanced oxygen reduction catalysts. Appl. Catal. B Environ..

[74] Yang T., Liu J., Zhou R., Chen Z., Xu H., Qiao S.Z., Monteiro M.J. (2014). N-doped mesoporous carbon spheres as the oxygen reduction reaction catalysts. J. Mater. Chem. A.

[75] Kim S.Y., Suh W.H., Choi J.H., Yi Y.S., Lee S.K., Stucky G.D., Kang J.K. (2014). Template-free synthesis of high surface area nitrogen-rich carbon microporous spheres and their hydrogen uptake capacity. J. Mater. Chem. A.

[76] Liu Y., Jin X.J., Dionysiou D.D., Liu H., Huang Y.M. (2015). Homogeneous deposition-assisted synthesis of iron nitrogen composites on graphene as highly efficient non-precious metal electrocatalysts for microbial fuel cell power generation. J. Power Sources.

[77] Gong X.B., You S.J., Wang X.H., Zhang J.N., Gan Y., Ren N.Q. (2014). A novel stainless steel mesh/cobalt oxide hybrid electrode for efficient catalysis of oxygen reduction in a microbial fuel cell. Biosens. Bioelectron..

[78] Qiao Y., Wu X.-S., Ma C.-X., He H., Li C.M. (2014). A hierarchical porous graphene/nickel anode that simultaneously boosts the bio- and electro-catalysis for high-performance microbial fuel cells. RSC Adv..

[79] Wen Q., Wang S., Yan J., Cong L., Chen Y., Xi H. (2014). Porous nitrogen-doped carbon nanosheet on graphene as metal-free catalyst for oxygen reduction reaction in air-cathode microbial fuel cells. Bioelectrochemistry.

[80] Cheng S., Liu H., Logan B.E. (2006). Increased performance of single-chamber microbial fuel cells using an improved cathode structure. Electrochem. Commun..

[81] Li W.W., Yu H.Q., He Z. (2014). Towards sustainable wastewater treatment by using microbial fuel cells-centered technologies. Energy Environ. Sci..

[82] kumar G.G., Sarathi V.G., Nahm K.S. (2013). Recent advances and challenges in the anode architecture and their modifications for the applications of microbial fuel cells. Biosens. Bioelectron..

[83] Baranitharan E., Khan M.R., Prasad D.M.R., Teo W.F.A., Tan G.Y.A., Jose R. (2015). Effect of biofilm formation on the performance of microbial fuel cell for the treatment of palm oil mill effluent. Bioproc. Biosyst. Eng..

[84] Zhang Y., Mo G., Li X., Zhang W., Zhang J., Ye J., Huang X., Yu C. (2011). A graphene modified anode to improve the performance of microbial fuel cells. J. Power Sources.

[85] Chen M.Q., Zeng Y.X., Zhao Y.T., Yu M.H., Cheng F.L., Lu X.H., Tong Y.X. (2016). Monolithic three-dimensional graphene frameworks derived from inexpensive graphite paper as advanced anodes for microbial fuel cells. J. Mater. Chem. A.

[86] Tang J.H., Yuan Y., Liu T., Zhou S.G. (2015). High-capacity carbon-coated titanium dioxide core-shell nanoparticles modified three dimensional anodes for improved energy output in microbial fuel cells. J. Power Sources.

[87] Zhao Y.L., Wang C.H., Zhai Y., Zhang R.Q., Van Hove M.A. (2014). Selective adsorption of L-serine functional groups on the anatase TiO_2_(101) surface in benthic microbial fuel cells. Phys. Chem. Chem. Phys..

[88] Wang Y.Q., Huang H.X., Li B., Li W.S. (2015). Novelly developed three-dimensional carbon scaffold anodes from polyacrylonitrile for microbial fuel cells. J. Mater. Chem. A.

[89] Gadhamshetty V., Krishnamurthy A., Koratkar N. Ultrathin graphene coating for passivating microbial corrosion in microbial fuel cells. Proceedings of the 245th ACS National Meeting.

[90] Feng Y.J., Yang Q., Wang X., Logan B.E. (2010). Treatment of carbon fiber brush anodes for improving power generation in air-cathode microbial fuel cells. J. Power Sources.

[91] Wang Y., Li B., Zeng L., Cui D., Xiang X., Li W. (2013). Polyaniline/mesoporous tungsten trioxide composite as anode electrocatalyst for high-performance microbial fuel cells. Biosens. Bioelectron..

[92] Cheng S.A., Logan B.E. (2007). Ammonia treatment of carbon cloth anodes to enhance power generation of microbial fuel cells. Electrochem. Commun..

[93] Zhao F., Rahunen N., Varcoe J.R., Chandra A., Avignone-Rossa C., Thumser A.E., Slade R.C. (2008). Activated carbon cloth as anode for sulfate removal in a microbial fuel cell. Environ. Sci. Technol..

[94] Zhang J., Li J., Ye D.D., Zhu X., Liao Q., Zhang B.A. (2014). Tubular bamboo charcoal for anode in microbial fuel cells. J. Power Sources.

[95] Han T.H., Sawant S.Y., Hwang S.J., Cho M.H. (2016). Three-dimensional, highly porous N-doped carbon foam as microorganism propitious, efficient anode for high performance microbial fuel cell. RSC Adv..

[96] Goto Y., Yoshida N., Umeyama Y., Yamada T., Tero R., Hiraishi A. (2015). Enhancement of electricity production by graphene oxide in soil microbial fuel cells and plant microbial fuel cells. Front. Bioeng. Biotechnol..

[97] Zhao C.-E., Gai P., Song R., Zhang J., Zhu J.-J. (2015). Graphene/Au composites as an anode modifier for improving electricity generation in *Shewanella*-inoculated microbial fuel cells. Anal. Methods.

[98] Yuan H., He Z. (2015). Graphene-modified electrodes for enhancing the performance of microbial fuel cells. Nanoscale.

[99] Zhao C.E., Wu J.S., Ding Y.Z., Wang V.B., Zhang Y.D., Kjelleberg S., Loo J.S.C., Cao B., Zhang Q.C. (2015). Hybrid conducting biofilm with built-in bacteria for high-performance microbial fuel cells. ChemElectroChem.

[100] Wang G.M., Qian F., Saltikov C., Jiao Y.Q., Li Y. (2011). Microbial reduction of graphene oxide by *Shewanella*. Nano Res..

[101] Jain A., Gazzola G., Panzera A., Zanoni M., Marsii E. (2011). Visible spectroelectrochemical characterization of *Geobacter sulfurreducens* biofilms on optically transparent indium tin oxide electrode. Electrochim. Acta.

[102] Huang L., Li X., Ren Y., Wang X. (2016). A monolithic three-dimensional macroporous graphene anode with low cost for high performance microbial fuel cells. RSC Adv..

[103] Sruti A.N., Jagannadham K. (2010). Electrical conductivity of graphene composites with In and In-Ga alloy. J. Electron. Mater..

[104] Tang J., Chen S., Yuan Y., Cai X., Zhou S. (2015). In situ formation of graphene layers on graphite surfaces for efficient anodes of microbial fuel cells. Biosens. Bioelectron..

[105] Wang L., Lu X.P., Lei S.B., Song Y.H. (2014). Graphene-based polyaniline nanocomposites: Preparation, properties and applications. J. Mater. Chem. A.

[106] Liu D.Q., Jia Z., Wang D.L. (2016). Preparation of hierarchically porous carbon nanosheet composites with graphene conductive scaffolds for supercapacitors: An electrostatic-assistant fabrication strategy. Carbon.

[107] Lee C., Wei X.D., Kysar J.W., Hone J. (2008). Measurement of the elastic properties and intrinsic strength of monolayer graphene. Science.

[108] Luo J., Lai J.P., Zhang N., Liu Y.B., Liu R., Liu X.Y. (2016). Tannic acid induced self-assembly of three-dimensional graphene with good adsorption and antibacterial properties. ACS Sustain. Chem. Eng..

[109] Wang H., Wang G., Ling Y., Qian F., Song Y., Lu X., Chen S., Tong Y., Li Y. (2013). High power density microbial fuel cell with flexible 3D graphene-nickel foam as anode. Nanoscale.

[110] Najafabadi A.T., Ng N., Gyenge E. (2016). Electrochemically exfoliated graphene anodes with enhanced biocurrent production in single-chamber air-breathing microbial fuel cells. Biosens. Bioelectron..

[111] Wang Z.J., Cao C.L., Zheng Y., Chen S.L., Zhao F. (2014). Abiotic oxygen reduction reaction catalysts used in microbial fuel cells. Chemelectrochemstry.

[112] Ben Liew K., Daud W.R.W., Ghasemi M., Leong J.X., Lim W.S., Ismail M. (2014). Non-Pt catalyst as oxygen reduction reaction in microbial fuel cells: A review. Int. J. Hydrogen Energy.

[113] Khilari S., Pandit S., Ghangrekar M.M., Das D., Pradhan D. (2013). Graphene supported alpha-MnO_2_ nanotubes as a cathode catalyst for improved power generation and wastewater treatment in single-chambered microbial fuel cells. RSC Adv..

[114] Lu Z.J., Bao S.J., Gou Y.T., Cai C.J., Ji C.C., Xu M.W., Song J., Wang R.Y. (2013). Nitrogen-doped reduced-graphene oxide as an efficient metal-free electrocatalyst for oxygen reduction in fuel cells. RSC Adv..

[115] Qu L.T., Liu Y., Baek J.B., Dai L.M. (2010). Nitrogen-doped graphene as efficient metal-free electrocatalyst for oxygen reduction in fuel cells. ACS Nano.

[116] Kannan M.V., Kumar G.G. (2016). Current status, key challenges and its solutions in the design and development of graphene based ORR catalysts for the microbial fuel cell applications. Biosens. Bioelectron..

[117] Lai L., Potts J.R., Zhan D., Wang L., Poh C.K., Tang C., Gong H., Shen Z., Lin J., Ruoff R.S. (2012). Exploration of the active center structure of nitrogen-doped graphene-based catalysts for oxygen reduction reaction. Energy Environ. Sci..

[118] Yu D., Zhang Q., Dai L. (2010). Highly efficient metal-free growth of nitrogen-doped single-walled carbon nanotubes on plasma-etched substrates for oxygen reduction. J. Am. Chem. Soc..

[119] Li S.Z., Hu Y.Y., Xu Q., Sun J., Hou B., Zhang Y.P. (2012). Iron- and nitrogen-functionalized graphene as a non-precious metal catalyst for enhanced oxygen reduction in an air-cathode microbial fuel cell. J. Power Sources.

[120] Feng Y., Shi X., Wang X., Lee H., Liu J., Qu Y., He W., Kumar S.M.S., Kim B.H., Ren N. (2012). Effects of sulfide on microbial fuel cells with platinum and nitrogen-doped carbon powder cathodes. Biosens. Bioelectron..

[121] Chatenet M., Genies-Bultel L., Aurousseau M., Durand R., Andolfatto F. (2002). Oxygen reduction on silver catalysts in solutions containing various concentrations of sodium hydroxide–comparison with platinum. J. Appl. Electrochem..

[122] Feng C., Lv Z., Yang X., Wei C. (2014). Anode modification with capacitive materials for a microbial fuel cell: An increase in transient power or stationary power. Phys. Chem. Chem. Phys..

[123] Dong H., Yu H., Wang X. (2012). Catalysis kinetics and porous analysis of rolling activated carbon-PTFE air-cathode in microbial fuel cells. Environ. Sci. Technol..

[124] Gangadharan P., Nambi I.M., Senthilnathan J. (2015). Liquid crystal polaroid glass electrode from e-waste for synchronized removal/recovery of Cr^+6^ from wastewater by microbial fuel cell. Bioresour. Technol..

[125] Woods M.P., Biddinger E.J., Matter P.H., Mirkelamoglu B., Ozkan U.S. (2010). Correlation between oxygen reduction reaction and oxidative dehydrogenation activities over nanostructured carbon catalysts. Catal. Lett..

[126] Mustakeem (2015). Electrode materials for microbial fuel cells: Nanomaterial approach. Mater. Renew. Sustain. Energy.

[127] Ci S., Cai P., Wen Z., Li J. (2015). Graphene-based electrode materials for microbial fuel cells. Sci. Chin. Mater..

[128] Dai Y., Chan Y., Jiang B., Wang L., Zou J., Pang K., Fu H. (2016). Bifunctional Ag/Fe/N/C catalysts for enhancing oxygen reduction via cathodic biofilm inhibition in microbial fuel cells. ACS Appl. Mater. Interfaces.

[129] Khomenko V.G., Barsukov V.Z., Katashinskii A.S. (2005). The catalytic activity of conducting polymers toward oxygen reduction. Electrochim. Acta.

[130] Liu Y., Li K., Liu Y., Pu L., Chen Z., Deng S. (2015). The high-performance and mechanism of P-doped activated carbon as a catalyst for air-cathode microbial fuel cells. J. Mater. Chem. A.

[131] Jiang H., Halverson L.J., Dong L. (2015). A miniature microbial fuel cell with conducting nanofibers-based 3D porous biofilm. J. Micromech. Microeng..

[132] Ren H., Lee H.S., Chae J. (2012). Miniaturizing microbial fuel cells for potential portable power sources: Promises and challenges. Microfluid. Nanofluid..

[133] He C.S., Mu Z.X., Yang H.Y., Wang Y.Z., Mu Y., Yu H.Q. (2015). Electron acceptors for energy generation in microbial fuel cells fed with wastewaters: A mini-review. Chemosphere.

[134] Lu M., Li S.F.Y. (2012). Cathode reactions and applications in microbial fuel cells: A review. Crit. Rev. Environ. Sci. Technol..

[135] Yan Z.H., Wang M., Huang B.X., Liu R.M., Zhao J.S. (2013). Graphene Supported Pt-Co alloy nanoparticles as Cathode Catalyst for Microbial Fuel Cells. Int. J. Electrochem. Sci..

[136] Liu X., Yang C., Zhang L., Li L., Liu S., Yu J., You L., Zhou D., Xia C., Zhao J. (2011). Metabolic profiling of cadmium-induced effects in one pioneer intertidal halophyte Suaeda salsa by NMR-based metabolomics. Ecotoxicology.

[137] Lee D.J., Chang J.S., Lai J.Y. (2015). Microalgae-microbial fuel cell: A mini review. Bioresour. Technol..

[138] Zhang G., Jiao Y., Lee D.J. (2015). A lab-scale anoxic/oxic-bioelectrochemical reactor for leachate treatments. Bioresour. Technol..

[139] Sharma V., Kundu P.P. (2010). Biocatalysts in microbial fuel cells. Enzyme Microb. Technol..

[140] Li R., Dai Y., Chen B., Zou J., Jiang B., Fu H. (2016). Nitrogen-doped Co/Co_9_S_8_/partly-graphitized carbon as durable catalysts for oxygen reduction in microbial fuel cells. J. Power Sources.

[141] Hou Y., Yuan H., Wen Z., Cui S., Guo X., He Z., Chen J. (2016). Nitrogen-doped graphene/CoNi alloy encased within bamboo-like carbon nanotube hybrids as cathode catalysts in microbial fuel cells. J. Power Sources.

[142] Ci S.Q., Wu Y.M., Zou J.P., Tang L.H., Luo S.L., Li J.H., Wen Z.H. (2012). Nitrogen-doped graphene nanosheets as high efficient catalysts for oxygen reduction reaction. Chin. Sci. Bull..

[143] Ansari M.O., Khan M.M., Ansari S.A., Amal I., Lee J., Cho M.H. (2014). pTSA doped conducting graphene/polyaniline nanocomposite fibers: Thermoelectric behavior and electrode analysis. Chem. Eng. J..

[144] Xu L., Zhang G.Q., Chen J., Yuan G.E., Fu L., Yang F.L. (2016). Prussian blue/graphene-modified electrode used as a novel oxygen reduction cathode in microbial fuel cell. J. Taiwan Inst. Chem. Eng..

[145] Li Y., Liu L., Yang F., Ren N. (2015). Performance of carbon fiber cathode membrane with C–Mn–Fe–O catalyst in MBR–MFC for wastewater treatment. J. Membr. Sci..

[146] Wang H., Park J.D., Ren Z.J. (2015). Practical energy harvesting for microbial fuel cells. Environ. Sci. Technol..

